# Physicochemical Properties and In Vivo Hepatoprotective Effect of Polysaccharides from Grape Pomace

**DOI:** 10.3390/antiox12020394

**Published:** 2023-02-06

**Authors:** Wenjun Miao, Rong Huang, Xiaoli Huang, Fei Gao, Xiangpeng Leng, Qiu Li

**Affiliations:** 1 Agricultural Bio-Pharmaceutical Laboratory, College of Chemistry and Pharmaceutical Sciences, Qingdao Agricultural University, Qingdao 266109, China; 2 Instrumental Analysis Center, Qingdao Agricultural University, Qingdao 266109, China; 3 Engineering Laboratory of Genetic Improvement of Horticultural Crops of Shandong Province, Institute of Grape Science and Engineering, College of Horticulture, Qingdao Agricultural University, Qingdao 266109, China

**Keywords:** grape pomace, polysaccharide, antioxidant, hepatoprotection

## Abstract

Here, the polysaccharides from grape pomace, a by-product in the wine industry, were characterized and evaluated in vitro and in vivo. The polysaccharides were extracted and studied using spectroscopic and chemical methods. The results revealed that GPPs are rich in arabinose, galactose and glucuronic acid and are heteropolysaccharides without protein and nucleic acid, containing α-glycoside bonds with irregular clusters on the surface. In vitro antioxidant activity assays indicated that GPPs have concentration-dependent antioxidant activity. In vivo, GPPs markedly decreased the levels of TNF-a, IL-6, ALT, AST and MDA in serum and liver tissues and restored the levels of SOD, CAT and GSH. Additionally, further histopathological examination confirmed that GPPs could mitigate the injury of liver induced by CCl_4_. Our results demonstrate that GPPs had antioxidant and hepatoprotective effects, and they are expected to be a potential ingredient for functional foods or hepatoprotective drugs.

## 1. Introduction

Nowadays, the total output of grapes in the world is about 23.38 million tons, ranking in the top five among all fruits [1]. However, lots of solid residues (about 25% of the weight of grapes) are produced in the brewing process, and the solid residues are mainly composed of grape pomace [2]. Grape pomace mainly consists of grape skin (38~52%) and grape seed, which are rich in bioactive compounds such as phenols [3,4], oils [5], polysaccharides [6], proteins and dietary fibers [7]. These solid residues are usually discharged into the environment as waste, which causes more serious environmental pollution, such as surface and ground water pollution, waste decomposition and foul odor [8]. Therefore, the reuse of grape pomace could reduce the environmental impact, which greatly encourages wine industry sustainable growth and is particularly appealing to the food and pharmaceutical business.

In recent years, as natural biomolecules, plant polysaccharides have become a research hotspot with their low toxicity, safety, high efficiency and other characteristics [9,10,11,12,13]. Many natural polysaccharides have been proved to be crucial to free radicals to protect the body against oxidative damage [14,15,16,17]. Usually, grapes contain powerful antioxidants known as polyphenols. These are thought to have anti-inflammatory and antioxidant properties. Other compounds, such as resveratrol, lutein and zeaxanthin, also benefit health. Regarding the polysaccharides from grapes, it was reported that Japanese grape (Hovenia dulcis) polysaccharides can be exploited as a multi-functional additive or antioxidant agent [18]. Wine grape polysaccharides play important roles in the technological and sensory characteristics of wines. Moreover, many studies have mainly focused on the pharmacologic actions of grapes, and few studies have focused on the polysaccharides extracted from grapes and by-products, let alone their structural characterization and biological activities.

Inspired by research on grape ingredients as antioxidant agents, we set out to explore the value of grape pomace polysaccharides (GPPs). We wanted to know whether GPPs have antioxidant activity in vitro and in vivo. Therefore, in this study, we extracted polysaccharides from grape pomace and characterized the physicochemical properties. Then, the antioxidant activity of grape pomace polysaccharides was observed using oxidative tests in vitro, and the hepatoprotective effect on acute CCl_4_-caused liver damage in mice was tested in vivo.

## 2. Materials and Methods

### 2.1. Materials and Reagents

Grape pomace of Cabernet Sauvignon was collected from Table Grape Research Institute of Shandong Province, Qingdao, China. Macroporous resin AB-8 was purchased from Solarbio Technology Co., LTD. (Beijing, China). Standard monosaccharides (fucose rhamnose, arabinose, galactose, glucose, xylose, mannose, fructose, ribose, galacturonic acid, glucuronic acid, mannuronic acid and guluronic acid) were obtained from Sigma Chemicals Company (St. Louis, MI, USA). The diagnostic kits for inflammatory (TNF-α and IL-6) and antioxidant indicators (ALT, AST, SOD, CAT and GSH), and hepatotoxicity indexes (MDA) were from Meimian Biotechnology Co., LTD (Yancheng, China). All chemicals were of analytical grade and used without further purification.

### 2.2. Extraction and Purification of GPPs

The extraction of GPPs was prepared using the method of previous reports [19]. Briefly, we soaked the grape pomace sample in petroleum ether for degreasing and decolorized it with 95% ethanol for 4 h. The grape pomace sample was immersed in water at 85 °C for 2 h, and the supernatant was extracted after 10 min of centrifugation at 4000 rpm. Then, we concentrated the supernatant and added 4 volumes of absolute ethanol to precipitate overnight. After the precipitate was collected and dissolved, the solution was deproteinized with sevage reagent, and the treatment was repeated until no protein was separated from the two-phase layer. After that, AB-8 macroporous resin was added, and the sample was oscillated in a thermostatic oscillator for 16 h. The filtrate was extracted and repeatedly decolorized into a transparent liquid. The liquid was ultrafiltered with an ultrafiltration machine to remove small-molecule pigments and other substances, and the molecular weight was cut off to 10–100 kDa.

### 2.3. Experimental Design for Optimization

To determine the effect of liquid-to-solid ratio, ultrasound power and extraction time on the extraction yield of GPPs, further experiments were carried out on these factors using a single-factor design. In short, the liquid-to-solid ratio was assigned values from 1:1 to 5:1; ultrasound power ranged from 100 to 500; and the extraction time was set from 10 min to 50 min. During the optimization of the experimental factors, one factor was changed per test, while the other factors were kept constant. Response surface methodology (RSM), which was used to investigate the effects of the three variables, was utilized in accordance with the findings of the single-factor experiment.

### 2.4. Characterization of GPPs

#### 2.4.1. Molecular Weight Determination

The homogeneity of GPPs was detected with an HPLC system consisting of a waters e2695 platform (Waters, Milford, MA, USA) linked with an RI detector. The column was eluted using ultrapure water, and the flow rate was 0.6 mL/min using a TSK-gel G4000PWxl column (Tosho, Tokyo, Japan). The average molecular weight was detected using a calibration curve, which was established using Dextran standards (known molecular weights: 11,600, 48,600, 80,900, 147,600, 273,000, 667,800, 1,185,000 and 2,150,000).

#### 2.4.2. UV and Fourier Transform Infrared Spectrometry (FT-IR) Analyses

The polysaccharide sample was dissolved in distilled water, and distilled water was used as blank control. The UV spectrum was measured in the range of 200-800 nm with a UV-3900 UV spectrophotometer (Hitachi, Tokyo, Japan) to detect proteins and nucleic acids. The purified powder sample (2 mg) was dried and mixed with KBr (100 mg) powder evenly, and the tablet was pressed for Fourier transform infrared spectrometry (Thermo Nicolet, Waltham, MA, USA) in a scan range of 500–4000 cm^−1^.

#### 2.4.3. Determination of Monosaccharide Composition

As previously described [20,21], the monosaccharide components of GPPs were identified using ion chromatography. In brief, GPPs were dissolved into TFA acid (1 ml 2M); then, they were heated at 121 °C for 2 h and dried with a nitrogen blowing instrument [22]. Subsequently, we added methanol for cleaning; then, we blow-dried the sample and repeated methanol cleaning 2–3 times. We added sterile water to dissolve it and then transferred it to a chromatographic bottle for detection. The instrument parameters were as follows: Dionex ™ CarboPac ™ PA20 (150 × 3.0 mm, 10 μm) liquid chromatographic column; injection volume, 5 μL; mobile phase A (H_2_O); mobile phase B (0.1M NaOH); mobile phase C (0.1 M NaOH and 0.2M NaAc); flow rate, 0.5 mL/min; column temperature, 30 °C.

#### 2.4.4. SEM Analysis

We used a cotton swab to fasten the GPP sample and sprayed gold coating on the sample surface by means of ion sputtering. The microstructure of the purified fractions was obtained using a scanning electron microscope (JSM-IT500, JEOL, Tokyo, Japan) by magnifying the samples at 400× and 5000×.

### 2.5. Antioxidant Properties

#### 2.5.1. DPPH Radical Scavenging Assay

We detected DPPH radical scavenging activity according to the reported methods with minor modifications [23,24]. In brief, polysaccharide samples at different concentrations were diluted with distilled water. The sample group consisted of an equal volume mixture of 0.1 mM DPPH solution and GPP sample solution in a 96-well plate, ascorbic acid (VC) as positive control and absolute ethanol as blank control. Absorbance was measured at 517 nm. DPPH radical scavenging activity was calculated with the following formula: DPPH scavenging activity (%) = [1 − (A_1_ − A_0_)]/A_2_ × 100%, where A_0_—absorbance without sample; A_1_—absorbance of the added sample; and A_2_—absorbance of distilled water.

#### 2.5.2. ABTS Radical Scavenging Activity

According to the previously reported method [25], 7.4 mmol/L ABTS and 2.6 mmol/L K_2_S_2_O_8_ were mixed at 1:1 by volume, kept in the dark for 12 h at room temperature and diluted 40–50 times with absolute ethanol at 734 nm to measure the absorbance value of 0.7 ± 0.02. GPP samples at different concentrations (0, 0.625, 1.25, 2.5, 5 and 10 mg/mL) were mixed with ABTS working solution in a volume of 1:4, shaken for 10 s and left in the dark for 6 min. VC and distilled water were used as positive and blank controls, respectively, and the absorbance of the samples was measured in a 96-well plate at 734 nm. The cleaning effect was calculated as follows: ABTS scavenging activity (%) = (1 − A_1_/A_0_) × 100%, where A_0_—absorbance without sample; and A_1_—absorbance of the added sample.

#### 2.5.3. Hydroxyl Radical Scavenging Activity

According to the previously reported method [26], 1 mmol/L ferrous sulfate solution (FeSO_4_), 9 mmol/L hydrogen peroxide solution (H_2_O_2_) and 3 mmol/L salicylic acid–ethanol solution were successively added to GPPs at different concentrations (0, 0.625, 1.25, 2.5, 5 and 10 mg/mL). Then, the samples were allowed to react in a 37 °C constant temperature water bath for 30 min and cooled to room temperature, and the absorbance of the samples was measured at 510 nm. VC and distilled water were used as positive and blank controls, respectively, and the clearance rate was calculated as follows: OH^−^ scavenging activity (%) = [1 − (A_1_ − A_0_)]/A_2_ × 100%, where A_0_—absorbance without sample; A_1_—absorbance of the added sample; and A_2_—absorbance of distilled water.

### 2.6. Evaluation of In Vivo Hepatoprotective Effect of GPPs

#### 2.6.1. Animals and Treatment

As an important organ with metabolic function, the liver performs key detoxification functions in the metabolism of endogenous and exogenous substances [27]. A large number of studies have shown that oxidative stress is one of the pathological processes of liver injury [28,29,30]. Carbon tetrachloride (CCl_4_) can cause acute liver failure and is an effective hepatotoxic agent widely used in animal models [14,15]. The mechanism of CCl_4_-caused acute liver injury is the production of highly reactive trichloro-methyl radical and trichloromethyl peroxy radical through metabolism [16]. In order to explore the activity of grape pomace polysaccharides, a liver-injured murine model induced by CCl_4_ was established and used for evaluation. The study was conducted according to the guidelines of the Declaration of Helsinki and was approved by the Animal Care and Use Committee of Qingdao Agricultural University. The animal experiments followed the guidance of the ethics committee of Qingdao Agricultural University (code: IACUC-20220722-2). After 3 days of adaptive feeding under standard conditions (24 ± 2 °C, humidity at 50–60%, light–dark cycle time of 12/12 h, free access to water and standard pellet feed) [31], SPF ICR mice (obtained from Jinan Pengyue Laboratory Animal Breeding Co., LTD., Shandong, China) were randomly divided into 5 groups (*n* = 6, 3 males and 3 females; weight, 20 ± 2 g). Group I was the normal control group, Group III was the CCl_4_-induced acute liver injury model group; in the normal control group and the model group, the same amount of normal saline (10 mL/kg body weight per day) was intragastrically administered for 14 consecutive days. Group II was the positive control and received silymarin solution at the dose of 100 mg/kg body weight per day. Groups IV–V received GPP solution at different doses (100 and 200 mg/kg body weight per day, respectively) for 14 consecutive days. On the 15th day, all mice except for those of group I were intraperitoneally injected with olive oil solution containing 0.1% CCl_4_ (10 mL/kg body weight per day), whereas group I mice were intraperitoneally injected with the same volume of olive oil without CCl_4_. All mice were forced to fast but had free access to enough water for 16 h. All mice were then killed by means of blood extraction from the eyeballs, and the blood from the eyeballs was collected into sterile tubes. Blood samples were centrifuged (3000 r/min, 4 °C) for 10 min in order to acquire serum and stored at −80 °C. Livers were immediately obtained, dissected and rinsed with PBS solution [32]. Half of the liver was supplemented with 9 times the volume of PBS solution, and the liver tissue was ground. The supernatant from liver tissue was centrifuged at 5000 r/min and 4 °C for 15 min and stored at −80 °C for further analysis.

#### 2.6.2. Determination of Liver Index and Liver Appearance

Each mouse was weighed with an analytical balance before death, and liver weights were measured immediately after death. After weighing, mouse livers were placed on a white background plate for photo comparison and visual observation. The liver index formula used was Liver index = mean liver weight/mean body weight.

#### 2.6.3. Biochemical Analysis

Physiological and biological indexes in serum and liver homogenate of mice were determined with an ELISA kit. Using the manufacturer’s instructions, the contents of serum TNF-α, IL-6, ALT and AST and the activities of MDA, SOD, CAT and GSH in liver tissue homogenate were detected with an enzyme-linked immunosorbent assay (ELISA) kit from Jiangsu Meimian industrial Co., LTD (Taizhou, China).

#### 2.6.4. Histopathological Observation

The pathology of liver preserved in 4% paraformaldehyde, embedded in paraffin and cut into serial sections (5 μm) was analyzed [33]. Histopathological changes were observed with a microscope (Nikon Eclipse E100, Nikon, Tokyo, Japan), and staining with hematoxylin and eosin as well as Hoechst staining, respectively, was performed (×200 magnifications).

### 2.7. Statistical Analysis

Analyses were repeated at least three times, and data were expressed as means ± standard deviations. The results were analyzed and tested with one-way analysis of variance (ANOVA) and Graphpad Prism 8.0, respectively. All statistical analyses were conducted and run in SPSS (version 21.0).

## 3. Results and Discussion

### 3.1. Single-Factor Tests

**Figure 1:**
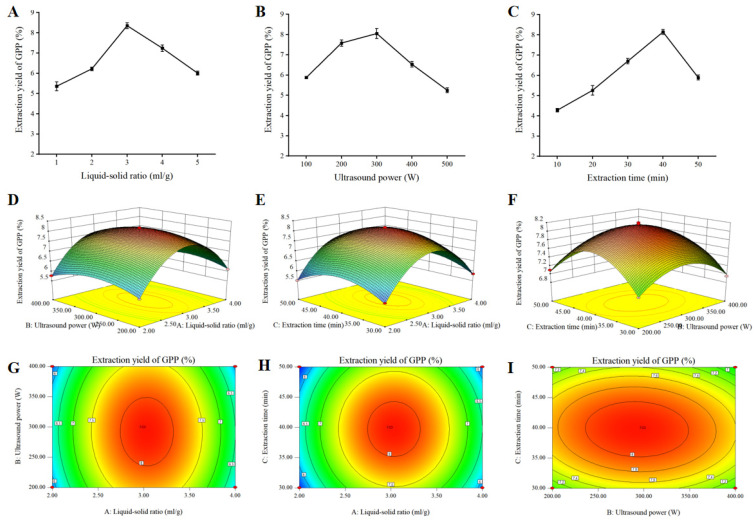
Single-factor experiment results and response surface plots showing the effects of interactions among different selected factors on the extraction yield of grape pomace polysaccharides (GPPs). (**A**–**C**) Effects of different liquid–solid ratio (**A**), ultrasound power (**B**) and extraction time (**C**) on the yield of GPPs in single-factor experiments. (**D**–**I**) Three- and two-dimensional plots showing the effects of liquid–solid ratio (**D**,**G**), ultrasound power (**E**,**H**) and extraction time (**F**,**I**) on extraction yield of GPPs, respectively.

In this study, we extracted a large amount of polysaccharides (GPPs) with high added value from low-value by-product grape pomace, which also provided a reference for the solution of the corresponding inedible waste reuse. The effects of different factors on the extraction yield of GPPs are shown in Figure 1A–C. The extraction efficiency reached a maximum value of 8.35% at 3:1 and then decreased as the liquid-to-solid ratio continued to increase (Figure 1A). With fixed liquid-to-solid ratio and extraction time at 3:1 and 40 °C, respectively, the extraction of GPPs increased as the ultrasound power was increased from 100 W to 300 W, reached a maximum at 300 W and then decreased as shown in Figure 1B. With fixed liquid-to-solid ratio and ultrasound power of 3:1 and 300 W, the extraction of GPPs reached a maximum at 40 min (Figure 1C). Table 1 displays the levels and codes of the extraction variables utilized in the Box–Behnken design (BBD).

### 3.2. Response Surface Analysis

Based on the findings of 17 random BBD test points, the encompassing design and experimental values are indicated in Table 2. The GPP extraction rate and projected response Y may be fitted to the following second-order polynomial equation using experimental data from multiple regression analysis:

Y = 8.14 + 0.12 × A − 0.071 × B − 0.039 × C − 0.023 × AB − 2.500 E − 003 × AC − 0.055 × BC − 1.76 × A^2^ − 0.46 × B^2^ − 0.71 × C^2^

where Y is the extraction yield of GPPs (%); and A, B and C are the liquid-to-solid ratios, ultrasound power and extraction time, respectively.

The ANOVA for this model is shown in Table 3. Lack of fit can be used to judge the validity of the model; as shown in Table 3, the lack-of-fit value of the mathematical model of 0.2766 was insignificant relative to the pure error. The high absolute F-value of 878.22 and the low *p*-value (*p* < 0.0001) indicated a significant model. Moreover, the high R^2^ score (R^2^ = 0.9991) suggested that this model could be adequately explained by 99.91% of the variability and accuracy of the response. Additionally, R^2^_Adj_ was 0.9980, which implied that the model was highly correlated with the experimental data and the theoretical values of extraction yield of GPPs. There was a relatively low value of the coefficient of variation (C.V. = 0.70%), which reflected high reproducibility and reliability of the experimental values.

In order to predict the relationship between independent variables and dependent variables, Design-Expert (version 8.0) software was used to establish the 3D response surface and contour maps as presented in Figure 1D–I. The interaction effects of liquid-to-solid ratio (A) and ultrasound power (B) are shown in Figure 1D,G, and the extraction yield increased with the increase in liquid-to-solid ratio and ultrasonic power. The combined effect of liquid-to-solid ratio (A) and extraction time (C) on the GPP yield is displayed in Figure 1E,H, where the steep surface indicates the significance of the effect between liquid-to-solid ratio (A) and extraction time (C). The effects of ultrasound power (B) and extraction time (B) are illustrated in Figure 1F,I. These results show significant effects of A, B and C on the extraction of GPPs. A liquid-to-solid ratio of 3.03:1, an ultrasound power of 292.47 W and an extraction time of 39.75 min were chosen as the optimal parameters.

### 3.3. Structural Characterization of GPPs

#### 3.3.1. UV and FT-IR of GPPs

**Figure 2:**
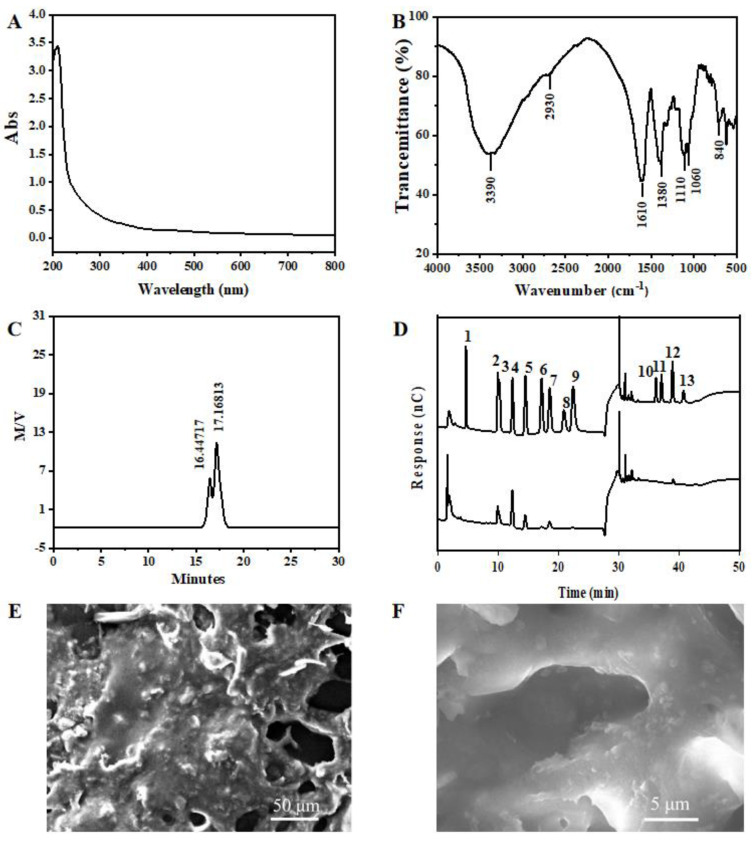
Structural characterization of grape pomace polysaccharides (GPPs). (**A**) UV spectrum of GPPs. (**B**) FT-IR spectrum of GPPs. (**C**) Molecular weight of GPPs. (**D**) Monosaccharide composition of GPPs; the standards from 1 to 13 were Fuc, Ara, Rha, Gal, Glc, Xyl, Man, Fru, Rib, GalA, GulA, GlcA and ManA, respectively. SEM images of grape pomace polysaccharides at (**E**) 400× and (**F**) 5000×, respectively.

GPPs with uniform composition were obtained by means of deproteinization, decolorization and ultrafiltration separation. The UV spectrum of GPPs is shown in Figure 2A. There are no special absorption peaks at 260 nm and 280 nm, which means that the nucleic acids or conjugated proteins were removed with GPP purification.

As shown in Figure 2B, the FT-IR spectrum of GPP at 4000~500 cm^−1^ shows the characteristic peaks of polysaccharides. The strong absorption bands at 3390 cm^−1^ and 2930 cm^−1^ represent the stretching vibration of O-H and C-H in sugar molecules, respectively. The broad and intense band detected near 1610 cm^−1^ is due to the stretching vibration of COO-, indicating the presence of uronic acid in the polysaccharide. The peak at 1380 cm^−1^ is attributed to the C-H bending vibration, and absorption between 800 and 1200 cm^−1^ is considered the fingerprint area that can reflect the vibration of the sugar ring. The two strong absorption bands observed at 1060 cm^−1^ and 1100 cm^−1^ are related to the C-O-C stretching of the glycosidic bond and confirmed that the main style of the monosaccharides in GPPs was the pyran type. In addition, absorption at 840 cm^−1^ and 816 cm^−1^ indicates the presence of α Type A glycosidic bonds.

#### 3.3.2. Molecular Weight and Monosaccharide Composition Analysis

The average molecular weight of the main GPP was 17.168 × 10^5^ Da (Figure 2C), according to the calibration curve with standards. After the analysis of the monosaccharide composition of this GPP using IC, as shown in Figure 2D, this GPP was found to be a heteropolysaccharide, mainly composed of Ara, Rha, Gal, Glc, Man and Glc-UA. The ratios of monosaccharides are listed in Table 4. The data show that Ara was the main monosaccharide, with the largest proportion of GPPs.

#### 3.3.3. SEM Analysis of GPPs

In order to observe the microstructure of GPPs, SEM imaging was performed. As shown in Figure 2E,F, at 400×, GPPs showed irregular lumpy aggregation and fine, bonded, convex particles on the surface. At 2000× magnification, more holes were observed between the clusters, which may have been due to the mutual repulsion of intermolecular attraction.

### 3.4. Antioxidant Activity Analysis

#### 3.4.1. DPPH Radical Scavenging Activity

**Figure 3:**
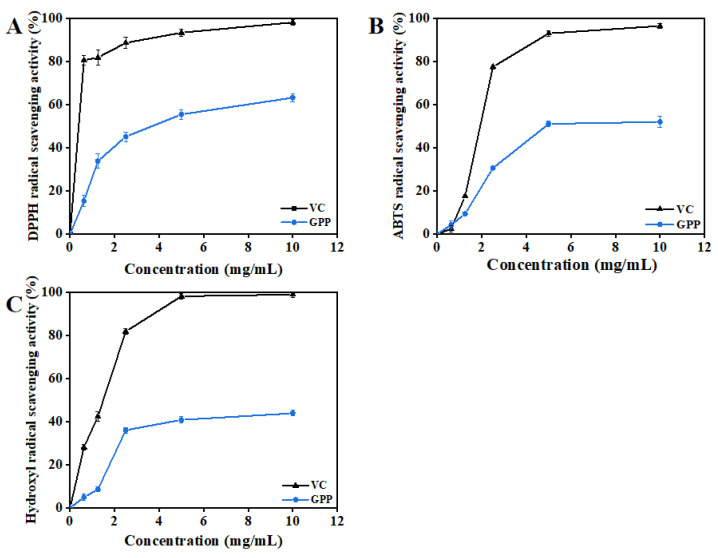
Antioxidant activity of grape pomace polysaccharides. (**A**) DPPH radical scavenging capacity, (**B**) ABTS radical scavenging capacity and (**C**) OH^−^ scavenging capacity. All treatments were performed in triplicate, and data are shown as means ± standard deviations. Ascorbic acid was the positive control.

DPPH radical scavenging activity has been widely used to evaluate the free radical scavenging activity of natural extracts and provides an important reflection of the screening of antioxidation activity [34]. The results in Figure 3A depict the DPPH radical scavenging activity of VC and GPPs at different concentrations. The highest inhibition was 63.23% at a dosage of 10 mg/mL and still below the same concentration of VC (98.18%). DPPH radical scavenging activity was observed to increase significantly with the increase in GPP concentration, indicating that the scavenging activity increased in a dose-dependent manner.

#### 3.4.2. ABTS Radical Scavenging Activity

Hydrogen atoms or electrons can be accepted via the reaction between ABTS radicals and antioxidants. It is a commonly used method to detect antioxidant capacity in vitro [35]. As shown in Figure 3B, it is clear that all samples exhibited ABTS radical scavenging in a significant concentration-dependent manner. At the maximum dose of 10 mg/mL, the values of the ABTS^+^ scavenging activity of GPPs and VC were 52.01% and 96.51%, respectively. Therefore, it could of great significance and value to extract GPPs and apply them as natural antioxidants in the food industry.

#### 3.4.3. Hydroxyl Radical Scavenging Activity

The Hydroxyl radical is considered the most dynamic and harmful free radical and can affect biological macromolecules in living cells [36]. As shown in Figure 3C, it is obvious that GPPs and VC showed significant concentration dependence at concentrations of 0~10.0 mg/mL. The maximum OH^−^ radical scavenging activity of GPPs was 43.96% at 10 mg/mL, whereas the lowest ABTS radical scavenging activity was 4.23% at 0.125 mg/mL. Moreover, 5 mg/mL VC could fully scavenge hydroxyl radicals. Based on the results of three kinds of antioxidant free radical removal, the high antioxidant activity of purified GPPs in vitro may have been due to their high uronic acid content, large molecular weight and abundant monosaccharide composition [37].

### 3.5. Evaluation of In Vivo Hepatoprotective Activity of GPPs

#### 3.5.1. Effects of Inflammatory Cytokines in Serum Induced by GPPs

**Figure 4:**
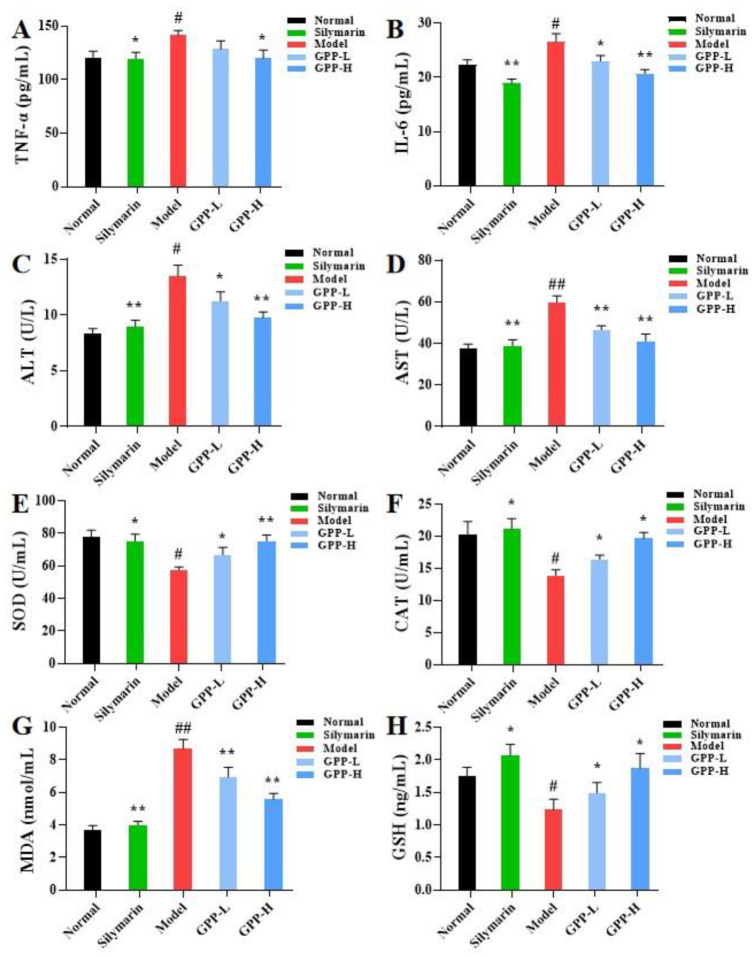
(**A**,**B**) The secretion of inflammatory cytokines (TNF-α and IL-6), (**C**–**H**) enzyme activity (ALT, AST, SOD, CAT and GSH) and lipid metabolite contents (MDA) in mice were measured. SOD, CAT, MDA and GSH in liver tissues were measured. Compared with the model group, * in the same column indicates significant difference (*p* < 0.05). Compared with the normal group, # means significant difference (*p* < 0.05). Values are presented as means ± SDs (*n* = 3).

In order to verify the effect of GPPs on the levels of inflammatory factors in CCl_4_-induced liver injury in mice, we measured the important inflammation-related indicators involved in normal inflammatory response and immune response, TNF-α and IL-6. As shown in Figure 4A,B, the levels of TNF-α and IL-6 in the model group were significantly increased (*p* < 0.05) when compared with the normal group, indicating that the model was successfully established. Nevertheless, pretreatment with GPPs significantly reduced the levels of TNF-α and IL-6 compared with the model group; expression of the GPP-H group was similar to that of the positive control group, showing that a high dose of GPP could alleviate liver inflammation caused by CCl_4_.

#### 3.5.2. Effects of GPPs on Serum ALT and AST Activities

Serum ALT and AST enzymatic activities have been regarded as evaluation indicators of liver injury. The levels of serum ALT and AST activities were measured in this work [38]. As shown in Figure 4C,D, serum hepatic enzymes (ALT and AST) were significantly increased in CCl_4_-induced liver injury compared with the control group, whereas compared with the model group, the silymarin group had significantly decreased contents of ALT and AST enzymes. Moreover, it was observed that the 100 mg/mL and 200 mg/mL GPP groups effectively prevented the CCl_4_-induced elevation in serum ALT and AST in a dose-dependent manner. GPP pretreatment at high dose (200 mg/kg body weight) decreased the serum ALT and AST levels to 9.75 ± 0.53% and 41.09 ± 3.48%, respectively, suggesting that GPPs could ameliorate the effects on liver injury induced by CCl_4_.

#### 3.5.3. Effects of GPPs on Oxidative Stress in Liver

The levels of SOD, CAT, GSH and MDA were determined with an ELISA kit to evaluate the oxidative stress state of CCl_4_-induced acute liver injury in mice [39]. As shown in Figure 4E–H, the injection of CCl_4_ could obviously reduce SOD, CAT and GSH activities (*p* < 0.05) and elevate MDA contents (*p* < 0.05) when compared with the normal control. Mice pretreated with GPPs showed significant improvement in concentration dependence. The high-dose (200 mg/kg body weight) GPP pretreatment increased the levels of SOD, CAT, MDA and GSH in liver by 31.24%, 41.73% and 57.18%, respectively, and decreased the MDA level by 1.6 times compared with the model group. These results demonstrate that GPP pretreatment was able to mitigate lipid peroxidation and possessed protective action on the oxidative damage of liver tissue caused by CCl_4_.

#### 3.5.4. Effects of GPPs on Morphology in Mice and Liver Index

**Figure 5:**
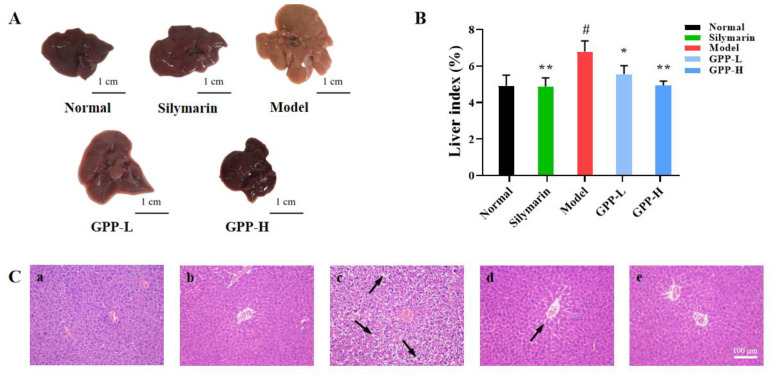
(**A**) Appearance of liver tissues. (**B**) Liver index of the different groups. (**C**) Histopathological evaluation of liver tissues, stained with H&E (200×): (a) normal group; (b) positive group, pretreatment with silymarin; (c) model group; (d,e) GPP-treated groups at different concentrations of GPPs (100 mg/kg and 200 mg/kg). The black arrow indicates hepatic cell necrosis and vacuolization. Compared with model group, * in the same column indicates significant difference (*p* < 0.05). Compared with the normal group, # means significant difference (*p* < 0.05). Values are presented as means ± SDs (*n* = 3).

CCl_4_-induced liver injury in vivo is related to the oxidative stress mechanism. In this study, the antioxidant effect was further verified using CCl_4_-induced acute liver injury in mice [40]. The main bioactive substances of silymarin are considered to have antioxidant activity [41,42]. As shown in Figure 5A, compared with the normal control, the liver of the CCl_4_ model group was enlarged, with vacuolar degeneration and fibrous hyperplasia, and the texture was rough. In the low-dose group, the texture became soft and dispersed. There was an insignificant difference among the high-dose group, positive control group and normal group. Compared with the model group, the prophylactic administration of GPPs at 100 and 200 mg/kg body weight concentrations could significantly reduce the liver index of mice (*p* < 0.05) (Figure 5B), and the liver index of the GPP-L and GPP-H groups significantly decreased to 5.56 ± 0.46% and 4.94 ± 0.23%, respectively. Interestingly, treatment with 200 mg/kg body weight alone showed no significant differences when comparing the normal control and positive control groups. The results demonstrate that a high dose (200 mg/kg body weight) of GPPs could improve the swelling and inflammatory infiltration of liver tissue induced by CCl_4_ and had a certain hepatoprotective effect.

#### 3.5.5. Histopathology of Liver

In order to confirm the protective effect of GPPs on liver injury, the histopathological changes of the liver were observed. As shown in Figure 5C, the liver sections of mice in the normal control group exhibited their normal histological features, with classical hepatic lobules. On the other hand, the CCl_4_ liver tissues of severe injury models were observed to have large area swelling and necrosis of liver cells, vacuolization, inflammatory cell infiltration, and other morphological and histological changes. In contrast, in the silymarin group and GPP groups, the vacuole area of liver cells was reduced, and the swelling and degeneration of liver cells, inflammatory infiltration and other pathological phenomena were significantly improved. In addition, the GPP groups showed significantly recovered histological lesions, and the results of the histopathological analyses were consistent with those of the biochemical analyses.

## 4. Conclusions

In this study, GPPs were isolated from grape pomace and characterized using a series of technologies. We found that GPPs contain a high content of arabinose and mannose, which might possess antioxidant activity [43,44]. To better determine the effect of GPPs, the antioxidant activity of GPPs in vitro was studied. The result show that GPPs exhibited potent antioxidant activity.

Free radicals produced by CCl_4_ in hepatocytes can lead to oxidative stress, loss of enzyme function and lipid peroxidation of the cell membrane, ultimately leading to liver damage [45]. To explore its further antioxidant ability, an in vivo study was performed. The results show that GPP-pretreated mice improved the liver morphology caused by CCl_4_. Studies also showed that GPPs can reduce oxidative stress and thus achieve liver protection, which is related to free radical scavenging and antioxidant activity. This finding is consistent with other reports in which CCl_4_ inhibited oxidative stress [46,47]. In addition, the expression of TNF-α and IL-6 was significantly increased after treatment with CCl_4_. However, GP pretreatment could significantly decrease the expression of inflammatory factors. The histopathological results further confirm that GPPs could reduce oxidative stress and inhibit inflammation. In sum, GPPs may be exploited as a new source of natural antioxidants with potential functional food value, and this study provides a theoretical foundation for the further investigation of GPPs as a functional food additive.

## Figures and Tables

**Table 1:** Factors and level values of Box–Behnken.

Variable	Coded Factor Level		
	−1	0	1
Liquid–solid ratio (mL/g) (A)	2	3	4
Ultrasound power (W) (B)	200	300	400
Extraction time (min) (C)	30	40	50

**Table 2:** Box–Behnken design (BBD) matrix of the three variables in coded units and response values for the extraction rate of GPPs.

Run	Liquid–Solid Ratio (A)	Ultrasonic Power (B)	Extraction Time (C)	Yield (%)
1	0	−1	−1	7.02
2	−1	1	0	5.77
3	0	−1	1	7.1
4	0	0	0	8.14
5	0	0	0	8.17
6	−1	0	1	5.5
7	0	1	1	6.81
8	1	0	1	5.72
9	1	−1	0	6.11
10	−1	−1	0	5.83
11	0	0	0	8.13
12	−1	0	−1	5.62
13	1	0	−1	5.85
14	0	0	0	8.08
15	0	1	−1	6.95
16	1	1	0	5.96
17	0	0	0	8.2

**Table 3:** Analysis of variance (ANOVA) of the quadratic model and lack of fit.

Source	Sum of Squares	Degree of Freedom	Mean Square	F-Value	*p*-Value	Significance
Model	17.5	9	1.94	878.22	<0.0001	***
A	0.11	1	0.11	47.8	0.0002	
B	0.041	1	0.041	18.35	0.0036	
C	0.012	1	0.012	5.43	0.0526	
AB	2.025E-003	1	2.025E-003	0.91	0.3707	
AC	2.500E-005	1	2.500E-005	0.011	0.9183	
BC	0.012	1	0.012	5.47	0.0520	
A^2^	13.07	1	13.07	5905.47	<0.0001	***
B^2^	0.91	1	0.91	410.41	<0.0001	***
C^2^	2.12	1	2.12	957.52	<0.0001	***
Residual	0.015	7	2.561E-003			
Lack of fit	7.375E-003	3	3.483E-003	1.86	0.2766	Not significant
Pure error	8.120E-003	4	1.870E-003			
Cor total	17.51	16				
R-squared	0.9991					
Adj R-squared	0.9980					
CV%	0.70%					
Pred R-squared	0.9825					
Adeq precision	72.683					

**Table 4:** Monosaccharide composition of grape pomace polysaccharides.

Sample	Percentage of Each Monosaccharide Group (%)					
	Ara	Rha	Gal	Glc	Man	Glc-UA
GPPs	20.79	9.63	18.67	18.76	16.09	7.53

## Data Availability

Data is contained within the article.
